# Targeting transcription of *MCL-1* sensitizes *HER2*-amplified breast cancers to HER2 inhibitors

**DOI:** 10.1038/s41419-021-03457-6

**Published:** 2021-02-15

**Authors:** Konstantinos V. Floros, Sheeba Jacob, Richard Kurupi, Carter K. Fairchild, Bin Hu, Madhavi Puchalapalli, Jennifer E. Koblinski, Mikhail G. Dozmorov, Sosipatros A. Boikos, Maurizio Scaltriti, Anthony C. Faber

**Affiliations:** 1 Department of Oral and Craniofacial Molecular Biology, Philips Institute for Oral Health Research, VCU School of Dentistry and Massey Cancer Center, Virginia Commonwealth University, Richmond, VA 23298 USA; 2 Department of Pathology, Virginia Commonwealth University School of Medicine and Massey Cancer Center, Richmond, VA 23220 USA; 3 Department of Biostatistics, Virginia Commonwealth University, Richmond, VA 23298 USA; 4 Division of Hematology, Oncology and Palliative Care, Virginia Commonwealth University and Massey Cancer Center, Richmond, VA 23298 USA; 5 AstraZeneca Pharmaceuticals, 35 Gatehouse Dr., Waltham, MA 02451 USA; 6 Human Oncology & Pathogenesis Program (HOPP), Memorial Sloan Kettering Cancer Center, New York, NY 10065 USA; 7 Department of Pathology, Memorial Sloan Kettering Cancer Center, New York, NY 10065 USA

**Keywords:** Breast cancer, Target identification

## Abstract

Human epidermal growth factor receptor 2 gene (*HER2*) is focally amplified in approximately 20% of breast cancers. HER2 inhibitors alone are not effective, and sensitizing agents will be necessary to move away from a reliance on heavily toxic chemotherapeutics. We recently demonstrated that the efficacy of HER2 inhibitors is mitigated by uniformly low levels of the myeloid cell leukemia 1 (MCL-1) endogenous inhibitor, NOXA. Emerging clinical data have demonstrated that clinically advanced cyclin-dependent kinase (CDK) inhibitors are effective MCL-1 inhibitors in patients, and, importantly, well tolerated. We, therefore, tested whether the CDK inhibitor, dinaciclib, could block MCL-1 in preclinical *HER2*-amplified breast cancer models and therefore sensitize these cancers to dual HER2/EGFR inhibitors neratinib and lapatinib, as well as to the novel selective HER2 inhibitor tucatinib. Indeed, we found dinaciclib suppresses *MCL-1* RNA and is highly effective at sensitizing HER2 inhibitors both in vitro and in vivo. This combination was tolerable in vivo. Mechanistically, liberating the effector BCL-2 protein, BAK, from MCL-1 results in robust apoptosis. Thus, clinically advanced CDK inhibitors may effectively combine with HER2 inhibitors and present a chemotherapy-free therapeutic strategy in *HER2*-amplified breast cancer, which can be tested immediately in the clinic.

## Introduction

HER2 inhibitors extend survival in *HER2*-amplified breast cancers; however, they are not sufficiently active as monotherapy^1,2^, unlike other receptor tyrosine kinase (RTK) inhibitors in solid tumor cancer paradigms. Due to this, there remains a reliance on chemotherapy; in contrast, in paradigms like epidermal growth factor receptor (*EGFR)-*mutant lung cancer and anaplastic lymphoma kinase (*ALK)*-translocated lung cancer, effective targeted therapy has mitigated the need of chemotherapy^3^.

We have demonstrated recently that *HER2*-amplified breast cancers have significantly lower NOXA levels, leading to MCL-1-mediated resistance to HER2 inhibitors through suppression of apoptosis^4^. Similarly, Merino et al.^5^ demonstrated that co-administration of MCL-1 inhibitors with HER2 inhibitors sensitizes *HER2*-amplified breast cancer models. While MCL-1 BH3 mimetics are advancing into clinical trials either alone or with venetoclax in hematological cancers, it remains uncertain whether these drugs will be able to sufficiently block the interaction of MCL-1 and proapoptotic BH3-only proteins such as NOXA and BIM. Moreover, the tolerability of these drugs in combination is unkown.

Inhibitors that block CDK9 can interfere with gene transcription. Thus, transcription of mRNAs with short half-lives that need to be synthesized at a high rate may be particularly affected by these agents^6^. Unique among the antiapoptotic proteins, MCL-1 has a very short half-life^7,8^. Dinaciclib has been used as an MCL-1 inhibitor in several cancer paradigms. It has already been reported that dinaciclib causes mitochondria-dependent apoptosis in osteosarcoma with MCL-1 being the primary target^9^, and in hepatocellular carcinoma dinaciclib decreases *MCL-1* mRNA levels without significantly changing the expression of other BCL-2 proteins^10^. Interestingly, CDK9 inhibition with dinaciclib is highly effective in MYC-driven lymphomas and involves downregulation of MCL-1^11^. And while there are also studies that support the elimination of MCL-1 at the protein level as the potential mechanism of action of dinaciclib^12^, most advocate for transcriptional downregulation of *MCL-1* as the critical mechanism^9,13^. In addition, we have recently demonstrated that the CDK inhibitor dinaciclib effectively blocks MCL-1 to sensitize EGFR inhibitors in *EGFR*-mutant non-small cell lung cancer (NSCLC)^14^. Dinaciclib exposure time peaks are roughly 2 h in humans, which is sufficient to block MCL-1, but not sufficient to block CDK1 or CDK2^15^. This suggests that the anticancer activity seen with dinaciclib is a result of its inhibitory effect on CDK9, and not CDK1/2. In a phase I trial in breast cancer patients, neutropenia and leukopenia were common, but dinaciclib in general was well tolerated^16^. In this study, we aimed to explore whether dinaciclib was sufficient to sensitize preclinical models of *HER2*-amplified breast cancer through downregulation of MCL-1.

## Results

### Dinaciclib sensitizes *HER2*-amplified breast cancers to HER2 inhibitors and is superior to the MCL-1 BH3 mimetic A-1210477

**Fig. 1:**
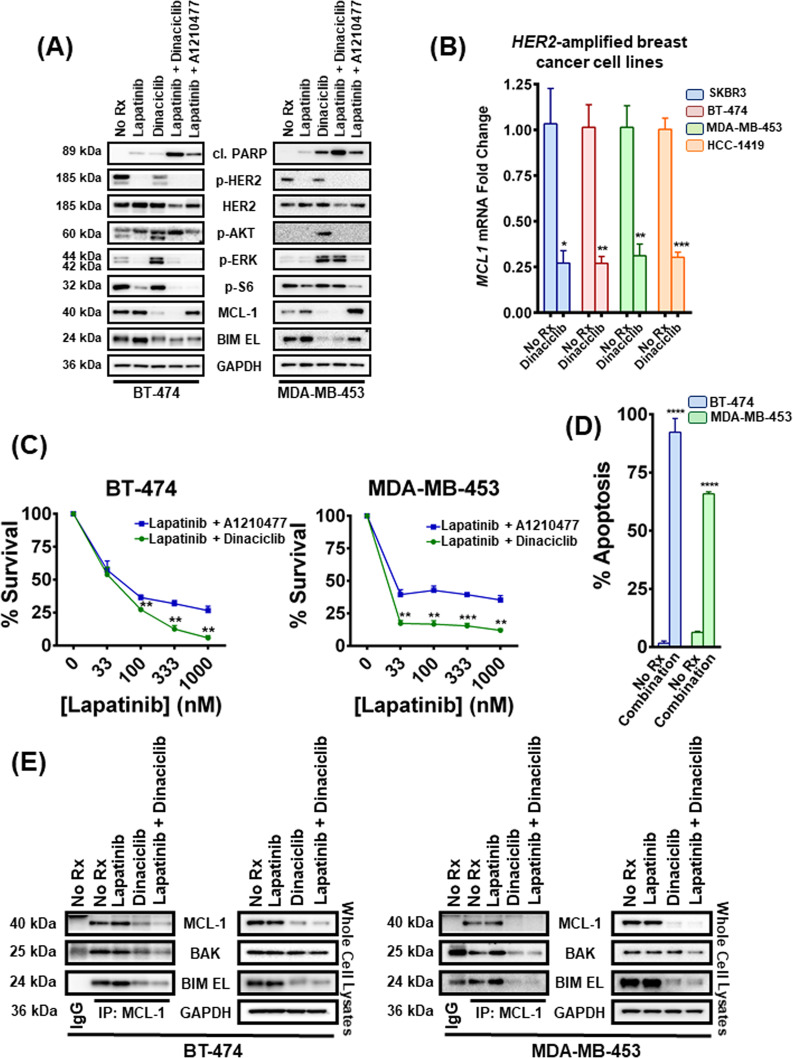
Dinaciclib sensitizes *HER2-*amplified breast cancer cells to lapatinib and liberates BAK from MCL-1. **A** BT-474 and MDA-MB-453 cells were treated with no drug, 1 μM lapatinib, 100 nM dinaciclib, their combination and the combination of 1 μM lapatinib with 10 μM A1210477 for 6 and 12 h, respectively. Whole-cell lysates were prepared, subjected to western blotting and probed for the indicated proteins. **B** Cells from SKBR3, BT-474, MDA-MB-453, and HCC-1419 *HER2*-amplified breast cancer cell lines were treated with no drug or 100 nM dinaciclib for 2 h, and levels of the abundance of *MCL-1* mRNA were analyzed by qPCR. Data are normalized to *ACTB*; *n* = 3; error bars indicate ±SEM. **C** BT-474 and MDA-MB-453 cells were treated with increasing concentrations of lapatinib and 10 μM A1210477 or with increasing concentrations of lapatinib and 100 nM dinaciclib for 24 and 72 h respectively, and the percentage of viable cells was determined. *n* = 3; error bars indicate ±SD. **D** BT-474 and MDA-MB-453 cells were treated with no drug or the combination of 1 μM lapatinib and 100 nM dinaciclib for 24 and 72 h, respectively and the percentage of annexin V/PI-positive cells was determined by FACS. *n* = 3, error bars indicate ±SD (“No Rx”: No drug). **E** MCL-1 complexes were immunoprecipitated from the indicated *HER2*-amplified breast cancer cell lines following 6 h (BT-474) and 12 h treatment (MDA-MB-453) with no drug, 1 μM lapatinib, 100 nM dinaciclib, and their combination. An IgG-matched isotype antibody was served as an immunoprecipitation control. The interaction between MCL-1 and BIM EL/BAK proteins was investigated (“No Rx”: No drug). For Fig. 1B–D two-tailed Student’s *t* test was performed. *p* values were corrected for multiple testing using the Bonferroni method. Differences were considered statistically different if *p* < 0.05. A *p* value < 0.05 is indicated by *, *p* < 0.01 by **, *p* < 0.001 by ***, *p* < 0.0001 by ****.

We and others recently demonstrated that pharmacological inhibitors of MCL-1 sensitized HER2 inhibitors in *HER2*-amplified breast cancers^4,5^. Based both on dinaciclib’s ability to inhibit MCL-1 in vitro and in vivo and its intrinsic therapeutic window, we investigated whether dinaciclib could be added to HER2 inhibitors and sensitize them through downregulation of MCL-1. In both *HER2*-amplified BT-474 and MDA-MB-453 cells, dinaciclib effectively reduced MCL-1 expression (Fig. 1A). In both cell lines, dinaciclib was more potent as a combining partner with the HER2 inhibitor lapatinib than was the MCL-1 BH3 mimetic A-1210477, as evidenced by cleaved PARP levels, a marker for apoptosis (Fig. 1A). In addition, while phosphorylation of HER2 was completely abolished, consistent with the on-target effect of lapatinib, HER2 levels were not significantly altered with any of the drug treatments (Fig. 1A). As expected, both the HER2/PI3K/TORC1 and HER2/RAS/TORC1 signaling pathways were disrupted by HER2 kinase inhibition, as evidenced by loss of pHER2, p-AKT (PI3K readout), p-ERK (RAS pathway readout), and p-S6 loss (mTORC1 pathway readout)^17^ (Fig. 1A). Dinaciclib strongly activated PI3K and MEK signaling, as evidenced by increased p-AKT (308) and p-ERK, respectively. However, lapatinib eventually abrogated both feedback activations (Fig. 1A). Of note, downregulation of MCL-1 by dinaciclib destabilizes also BIM EL (Fig. 1A), which was also noticed in our previous studies^4^.

In order to corroborate previous reports that dinaciclib-induced MCL-1 decreases are due to loss of MCL-1 transcription^10^, we evaluated *MCL-1* mRNA expression after treating different *HER2*-amplified breast cancer cell lines with dinaciclib (Fig. 1B). As expected, *MCL-1* mRNA expression was suppressed 2 h after dinaciclib addition. Consistently, after treating BT-474 cells for 24 h and the less sensitive MDA-MB-453 cells for 72 h, cell viability decreased more with the combination of lapatinib and dinaciclib than with lapatinib and A-1210477 (Fig. 1C). We further determined the sensitivity of the *HER2*-amplified breast cancer cell lines to the different combinations of these agents to gain information regarding the contribution of each single agent to the observed toxicity (Supplementary Fig. 1). In line with our previous data, dinaciclib displays a more synergistic potential with lapatinib than A-1210477 does. Altogether, these data indicate that dinaciclib downregulates MCL-1 and sensitizes to HER2 inhibitor in *HER2-*amplified breast cancers. Given that PARP cleavage has been reported to be implicated in other non-apoptotic processes^18,19^ and MCL-1 also exhibits apoptosis-independent functions in the cell^20,21^, we assessed Annexin V positivity by flow cytometry to confirm toxicity from loss of MCL-1 was due to an increase in apoptosis (Fig. 1D and Supplementary Fig. 2). To gain mechanistic insight, we immunoprecipitated MCL-1 in the BT-474 and MDA-MB-453 cells and observed that dinaciclib toxicity is mediated at least in part by BAK, which is liberated from MCL-1 following treatment and is free to execute its apoptotic program (Fig. 1E). Potential alterations in BIM EL:MCL-1 complexes were also investigated since BIM EL is a direct activator of Bcl-2-associated X protein (BAX)/Bcl-2 homologous antagonist/killer (BAK) molecules and its liberation could lead to further cell death responses. However, consistent with our previous data^22^, BIM EL levels were significantly downregulated in the whole-cell lysates following the addition of dinaciclib (Fig. 1E) making likely its role in combination toxicity, if any, limited.

### Dinaciclib sensitization to *HER2*-amplified breast cancers is abrogated by BAK knockdown and largely mediated by MCL-1

**Fig. 2:**
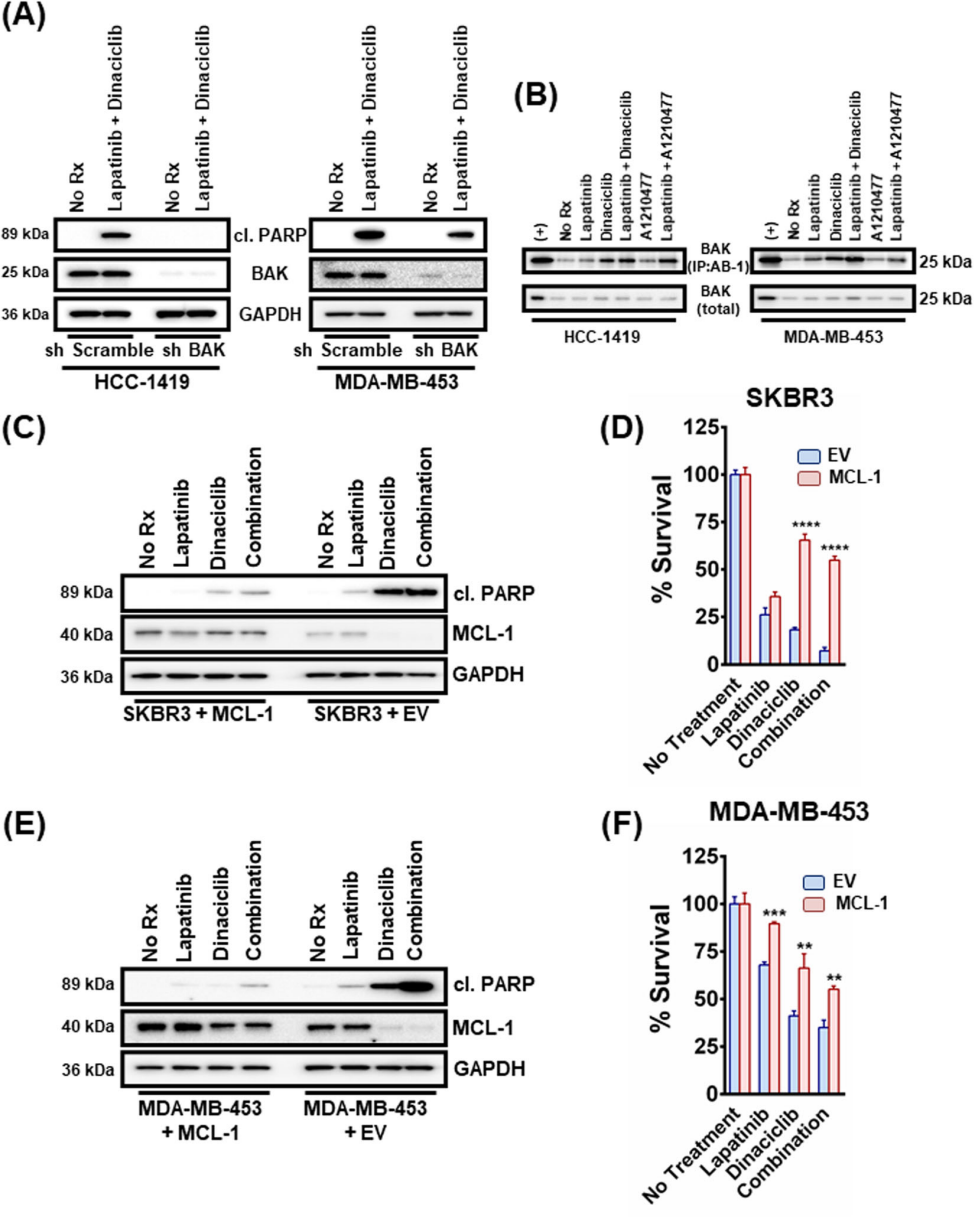
BAK is required for dinaciclib-induced cell death in *HER2*-amplified breast cancer cells and dinaciclib functions mainly by inhibiting MCL-1. **A** HCC-1419 and MDA-MB-453 cells were transduced with lentiviruses containing plasmids with an shRNA sequence targeting BAK or a non-targeting control. Puromycin-resistant cells were pooled after each infection. Cells were then treated with no drug, 1 μM lapatinib, 100 nM dinaciclib or their combination overnight. Cell lysates were prepared and subjected to western blotting and probed for cleaved PARP, BAK, and GAPDH (‘’No Rx”: No drug). **B** HCC-1419 and MDA-MB-453 cells were treated with no drug, 1 μM lapatinib, 100 nM dinaciclib, 10 μM A1210477 and their combinations (lapatinib/dinaciclib and lapatinib/A1210477) overnight and CHAPS lysates (using the zwitterionic detergent CHAPS, that can solubilize cells without promoting significant conformational changes in BAX and BAK, including the N-terminal Bak epitope exposure recognized by antibody Ab-1) were prepared and subjected to AB-1 IP and western blotting. Total cell lysates were analyzed in parallel. **C** SKBR3 control or MCL-1-expressing cells were treated with 1 μM lapatinib, 100 nM dinaciclib, and their combination for 12 h. Whole-cell lysates were prepared, subjected to western blotting and probed for the indicated proteins. **D** SKBR3 control or MCL-1-expressing cells were treated with 1 μM lapatinib, 100 nM dinaciclib, and their combination for 12 h and subjected to CellTiter-Glo. *n* = 3; error bars indicate ±SD. **E** MDA-MB-453 control or MCL-1-expressing cells were treated with 1 μM lapatinib, 100 nM dinaciclib, and their combination for 12 h. Whole-cell lysates were prepared, subjected to western blotting and probed for the indicated proteins. **F** MDA-MB-453 control or MCL-1-expressing cells were treated with 1 μM lapatinib, 100 nM dinaciclib and their combination for 72 h and subjected to CellTiter-Glo. *n* = 3; error bars indicate ±SD. For Fig. 2D, F two-tailed Student’s *t* test was performed. *p* values were corrected for multiple testing using the Bonferroni method. Differences were considered statistically different if *p* < 0.05. A *p* value < 0.05 is indicated by *, *p* < 0.01 by **, *p* < 0.001 by ***, and *p* < 0.0001 by ****. EV: empty vector, (+): positive control, CHAPS: 3- ((3-cholamidopropyl)dimethylammonio)-1-propanesulfonic acid.

As BAK-MCL-1 was sharply disrupted by dinaciclib, we sought to investigate this complex further and the role, if any, of BAK in dinaciclib and HER2 inhibitor/dinaciclib toxicity. Mechanistically, MCL-1 binds to BAK to prevent its activation^23^. Thus, if MCL-1 is critical to combination activity, BAK knockdown should mitigate the activity of the combination of dinaciclib and HER2 inhibition. Indeed, we found reduction of BAK by shRNA led to loss of apoptotic activity of the combination in two *HER2*-amplified HCC-1419 and MDA-MB-453 breast cancer cell lines where we were able to achieve sufficient knockdown (Fig. 2A). We next immunoprecipitated BAK with an antibody that exploits a conformation change in BAK upon its activation and only recognizes this active BAK species^24^. Consistent with an important role of MCL-1:BAK in combination toxicity, BAK was activated following either dinaciclib or A1210477 exposure, which was exacerbated upon the addition of lapatinib in both cases (Fig. 2B). Consistent with the enhanced apoptotic activity of the dinaciclib/lapatinib combination (Fig. 1A, B), BAK was more active following dinaciclib/lapatinib than A1210477/lapatinib therapy (Fig. 2B).

**Fig. 3:**
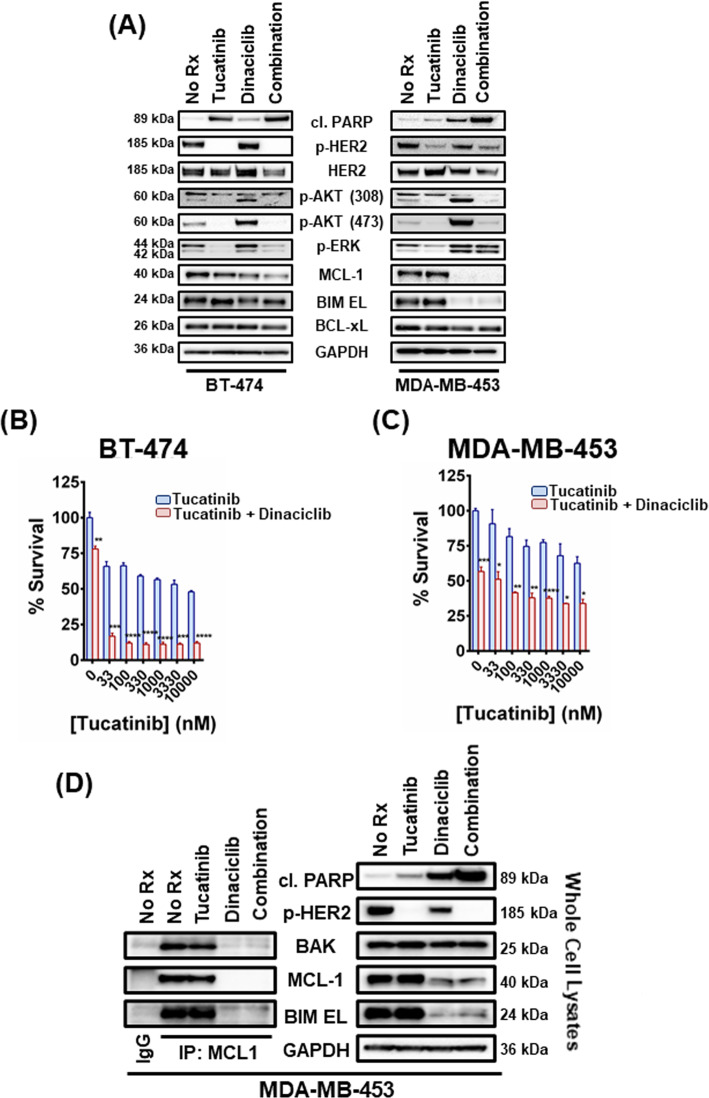
Dinaciclib sensitizes *HER2*-amplified breast cancer cells to tucatinib and liberates BAK from MCL-1. **A** BT-474 and MDA-MB-453 cells were treated with no drug, 1 μM tucatinib, 100 nM dinaciclib, and their combination for 6 and 12 h, respectively. Whole-cell lysates were prepared, subjected to western blotting and probed for the indicated proteins. **B** BT-474 cells were treated with increasing concentrations of tucatinib and 100 nM dinaciclib for 24 h and the percentage of viable cells was determined. *n* = 3; error bars indicate ±SD. **C** MDA-MB-453 cells were treated with increasing concentrations of tucatinib and 100 nM dinaciclib for 48 h and the percentage of viable cells was determined. *n* = 3; error bars indicate ±SD. **D** MCL-1 complexes were immunoprecipitated from MDA-MB-453 cells following 12 h treatment with no drug, 1 μM tucatinib, 100 nM dinaciclib, and their combination. An IgG-matched isotype antibody was served as an immunoprecipitation control. The interaction between MCL-1 and BIM EL/BAK proteins was investigated. For Fig. 3B, C two-tailed Student’s *t* test was performed; *p* values were corrected for multiple testing using the Bonferroni method. Differences were considered statistically different if *p* < 0.05. A *p* value < 0.05 is indicated by *, *p* < 0.01 by **, *p* < 0.001 by ***, and *p* < 0.0001 by ****. (“No Rx”: No drug).

**Fig. 4:**
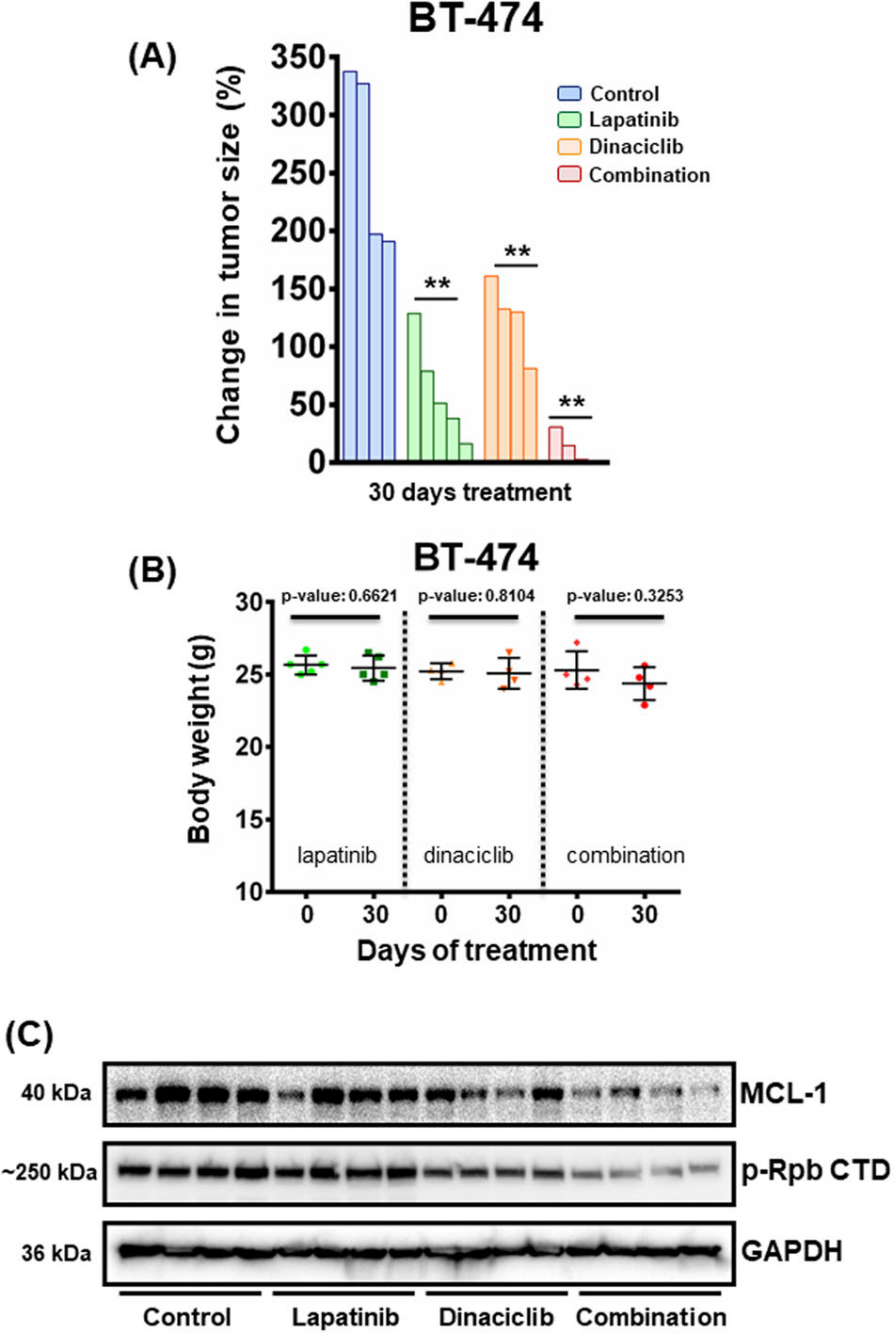
Combination treatment with lapatinib and dinaciclib leads to anti-tumor activity in vivo. **A** Approximately, 15 × 10^6^ (15 million) BT-474 cells were injected orthotopically into each NSG mouse (both sides) and monitored for subsequent growth. When tumors were ∼200 mm^3^, mice were randomized into treatment cohorts: control (no drug), 100 mg/kg lapatinib, 40 mg/kg dinaciclib, and their combination for 30 days. Dinaciclib was administered twice a week via IP injection. Lapatinib was given orally once a day for 5 consecutive days. Tumor measurements were performed daily, and the percentage (%) of changes in volume for each tumor is shown by a waterfall plot (control = 4 tumors, lapatinib = 5 tumors, dinaciclib = 4 tumors, combination = 4 tumors). For statistical analysis one-way Anova test was performed for comparisons between lapatinib, dinaciclib, and combination cohorts. Dunnett’s test was used as post hoc. Differences were considered statistically different if *p* < 0.05. A *p* value < 0.05 is indicated by *, *p* < 0.01 by **, *p* < 0.001 by ***, and *p* < 0.0001 by ****. **B** Weights of the single agents and the combination cohorts of the human xenograft-bearing mice. The number of mice was: control = 5 mice, lapatinib = 5 mice, dinaciclib = 4 mice, and combination = 4 mice. *p* values were calculated using the two-tailed Student’s *t* test. **C** Tumors were harvested from BT-474 tumor-bearing mice approximately 2 h after the last drug administration and tumor lysates were subjected to western blot analyses and probed for the indicated proteins.

While these data demonstrated a role of the MCL-1–BAK complex in dinaciclib/HER2 inhibitor combination efficacy, we sought to investigate how important the MCL-1–BAK complex was to combination efficacy. For these experiments, in addition to the MDA-MB-453 cells, we used the SKBR3 *HER2*-amplified breast cancer cell line, which is very sensitive to MCL-1 inhibition^4,25^. We found that the expression of exogenous MCL-1 was sufficient to mitigate the efficacy of both single-agent dinaciclib and the combination of dinaciclib and lapatinib to induce cell death (Fig. 2C), which translated into increased viability (Fig. 2D). In the MDA-MB-453 cells, rescue of MCL-1 expression was sufficient to block cell death (Fig. 2E) and increase total cell viability (Fig. 2F). To investigate if the other main pro-survival BCL2 proteins are implicated in dinaciclib-mediated apoptosis, we transiently overexpressed BCL2 and BCL-xL in the same two cell lines and treated with lapatinib, dinaciclib, and their combination (Suplementary Fig. 3 and Suplementary Fig. 4). Increased levels of BCL2 as well as BCL-xL did not result in significant suppression of the toxicity caused by the single agents or their combination, as determined by cleaved PARP expression (Suplementary Fig. 3A, C) or cell viability measurement (Suplementary Fig. 3B, D), demonstrating an MCL-1-specific effect caused by dinaciclib. However, while we did not see a sensitizing effect of the BCL-2 inhibitor venetoclax to lapatinib in the *HER2*-amplified breast cancer cell lines BT-474 or MDA-MB-453, we did see added toxicity with the tool BCL-xL inhibitor A-1331852, which was similar to that afforded by A-1210477 (Suplementary Fig. 4A, C). Similarly, A-1331852 sensitized the BT-474 and MDA-MB-453 cells to dinaciclib while venetoclax either did not (BT-474) or had a minimal effect (MDA-MB-453); strikingly, however, A-1210477 had no sensitizing effect on dinaciclib, consistent with MCL-1 as the key dinaciclib target in *HER2*-amplified breast cancer (Suplementary Fig. 4B, D).

### Dinaciclib sensitizes *HER2*-amplified breast cancer cells to the novel, selective HER2 inhibitor tucatinib

As there are now at least seven FDA-approved HER2 inhibitors^26^, we wanted to corroborate our findings with some of the newer HER2 inhibitors. Tucatinib is a novel, FDA-approved agent that has demonstrated more than 1000-fold selectivity for HER2 over EGFR in in vitro assays^27^ and significant efficacy in clinical trials for the treatment of metastatic *HER2*-positive breast cancer (NCT02614794)^28–32^. As expected from a HER2 inhibitor, tucatinib inhibited p-HER2, p-AKT and p-ERK in the *HER2*-amplified breast cancer cells BT-474 and MDA-MB-453^17^ (Fig. 3A). Addition of dinaciclib sensitizes the cancer cells to tucatinib as evidenced by increased cleaved PARP (Fig. 3A) and decreased cell viability in both cell lines (Fig. 3B, C), with their sensitivity reaching a plateau at about 1000 nM of tucatinib. To verify that complexes of MCL-1 with pro-apoptotic BCL2 proteins were disrupted by dinaciclib, we immunoprecipitated MCL-1 complexes in lysates derived from the MDA-MB-453 cells, following treatment with tucatinib, dinaciclib and their combination (Fig. 3D). Immunoprecipitation complex investigation confirmed that MCL-1:BAK complexes were disrupted following treatment with 100 nM dinaciclib (Fig. 3D).

### Dinaciclib is effective in vivo at sensitizing *HER2*-amplified breast cancers to HER2 inhibitors

**Fig. 5:**
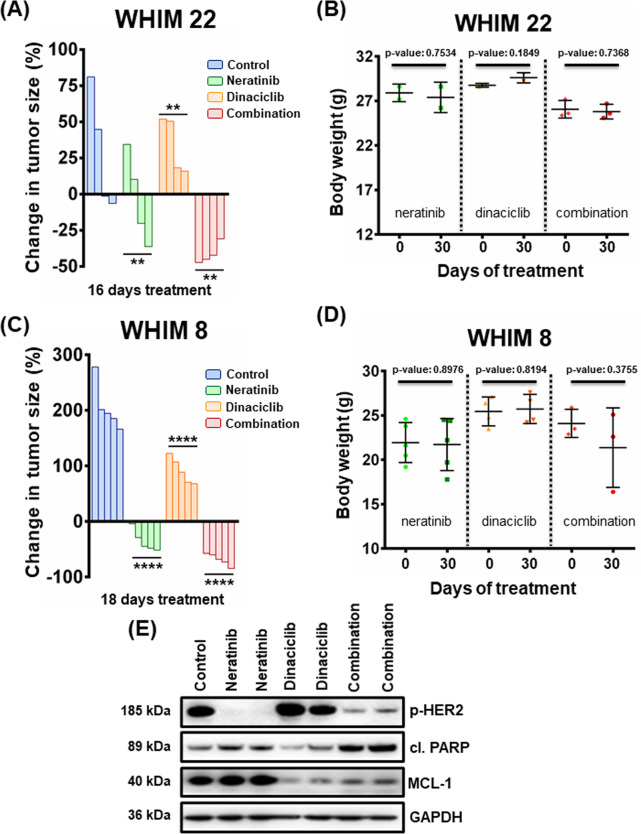
Combination treatment with neratinib and dinaciclib leads to anti-tumor activity in vivo. **A** Approximately, 1.5 × 10^6^ (1.5 million) cells derived from a *HER2*-positive breast cancer PDX model (WHIM 22) were injected orthotopically into each NSG mouse (both sides) and monitored for subsequent growth. After tumors reached a size of ~150 mm^3^, mice were treated with 40 mg/kg neratinib 5 days a week (Monday–Friday), 40 mg/kg dinaciclib twice a week, or their combination for 16 days. Tumor measurements were performed every day by calipers, and the percentage (%) of changes in volume for each tumor is shown by a waterfall plot (control = 4 tumors, neratinib = 4 tumors, dinaciclib = 4 tumors, combination = 4 tumors). For statistical analysis one-way Anova test was performed for comparisons between neratinib, dinaciclib, and combination cohorts. Dunnett’s test was used as post hoc. Differences were considered statistically different if *p* < 0.05. A *p* value < 0.05 is indicated by *, *p* < 0.01 by **, *p* < 0.001 by ***, and *p* < 0.0001 by ****. **B** Weights of the WHIM 22 PDX model-bearing mice of the single agents and the combination cohorts. The number of mice was: control = 2 mice, neratinib = 2 mice, dinaciclib = 2 mice, and combination = 3 mice. *p* Values were calculated using the two-tailed Student’s *t* test. **C** Same as **A** using the WHIM 8, *HER2-*positive breast cancer PDX model (18 days of treatment, control = 5 tumors, neratinib = 5 tumors, dinaciclib = 4 tumors, combination = 3 tumors). **D** Same as **B** using the WHIM 8 PDX model. The number of mice was: control = 5 mice, neratinib = 5 mice, dinaciclib = 2 mice, and combination = 3 mice. **E** Tumors were harvested from WHIM 8 PDX tumor-bearing mice approximately 2 h after the last drug administration and tumor lysates were subjected to western blot analyses and probed for the indicated proteins.

We next determined whether the combination of dinaciclib and lapatinib would be effective in vivo. As mentioned, exposure time, at least in humans, is sufficiently different and prevents the ability of dinaciclib to potently inhibit some CDK targets^15^. We found that dinaciclib exhibited modest efficacy when administered alone but was sufficient to significantly sensitize BT-474 xenografts to lapatinib when dosed twice a week based on the clinical schedule (Fig. 4A and Suplementary Fig. 5A). Mice remained healthy, based on their weight profiles, treated with the single agents or the combination (Fig. 4B). CDK9 phosphorylates the carboxy-terminal domain (CTD) of the RNA Polymerase II regulating elongation during transcription^33^. Thus, CDK9 inhibitors regulate the expression of proteins with a short half-life, like MCL-1, and the reduction of the phosphorylation of the RNA polymerase II CTD at Ser2 may be used as a biomarker of the activity of CDK9 inhibitors^34^. On-target inhibition of CDK9 was demonstrated by the suppression phosphorylation sites on the CTD of RNA polymerase II as well as MCL-1 following therapy with dinaciclib alone or in combination with lapatinib (Fig. 4C).

### Dinaciclib sensitizes neratinib in *HER2*-amplified patient-derived xenograft (PDX) models

Neratinib is a potent irreversible pan-HER inhibitor, recently FDA-approved for *HER2*-amplified breast cancer^2^. We tested neratinib in combination with dinaciclib in two *HER2*-amplified PDX models (WHIM 8 and WHIM 22)^35^. While neratinib was effective at blocking the growth of the *HER2*-amplified tumors, the combination of dinaciclib and neratinib was superior to single-agent therapy in the WHIM 22 model (Fig. 5A and Suplementary Fig, 5B). In addition, there was no weight loss of the mice treated with the single agents or the combination, again suggesting tolerability (Fig. 5B). In the WHIM 8 model, we observed high activity of neratinib monotherapy; however, the combination of neratinib and dinaciclib resulted in uniformly robust tumor shrinkage (>50%) (Fig. 5C and Suplementary Fig. 5C), with mice again not showing any significant weight loss (Fig. 5D). Cleaved PARP was elevated when the two drugs were administered together, indicating induction of apoptosis, while reduction of p-HER2 and MCL-1 advocates for the on-target effect of neratinib and dinaciclib, respectively (Fig. 5E). These data demonstrate potent combination efficacy of neratinib and dinaciclib in *HER2*-positive breast cancer PDX models.

## Discussion

HER2 inhibitors administered in the neo-adjuvant setting increase progression-free survival (the time from treatment initiation until disease progression or worsening) and overall survival (the duration of patient survival from the time of treatment initiation) in *HER2*-amplified breast cancers^36,37^. However, unlike similar RTK inhibitors in other solid tumor paradigms^38–40^ which have now replaced chemotherapy as standard of care, HER2 inhibitors are ineffective as monotherapy. Finding rational targeted therapy combinations with HER2 inhibitors therefore is likely the next step in order to find a therapeutic regimen that does not include chemotherapy.

Indeed, chemotherapy has already begun to be de-emphasized in breast cancer, in particular hormone positive breast cancer^41^. The reason for de-escalation is the broad and lasting effects of chemotherapy-induced toxicity, which has been well described in breast cancer. Toxicities are numerous and cover a wide range of tissues. Cardiac toxicity, including congestive heart failure, is contributed by anthracyclines like doxorubicin^42^. Reproductive toxicity is very common for breast cancer undergoing adjuvant chemotherapy: for instance, in 280 young (aged 24–45) breast cancer patients, over 90% suffered from chemotherapy-related amenorrhea^43^. While there remains controversy, a large Swedish study demonstrated women treated with chemotherapy for their breast cancer had higher risk pregnancies^44^. Chemotherapy-induced bone loss is also a significant toxicity with considerable morbidity^45,46^. In addition to overt tissue toxicity, chemotherapy delivered during breast cancer treatment increases the risk of secondary cancers, in particular acute myeloid leukemia^47,48^.

Recently, we reported that levels of the endogenous MCL-1 inhibitor, NOXA, are uniformly depressed in *HER2*-amplified breast cancers, as a result of a co-amplified intronic microRNA that targets the estrogen receptor (ER), which in turn leads to loss of ER-driven *NOXA* transcription^4^. This can be overcome by the addition of MCL-1 BH3 mimetics, which Merino et al.^5^ also demonstrated. However, the toxicity of these drugs in clinical trials remains to be defined. Interestingly, we also found co-targeting BCL-xL with HER2 is effective (Suplementary Fig. 4A, C), verifying results that have previously been reported^25^. In Fig. 2 and Suplementary Fig. 3B, D, we provide evidence that dinaciclib and consequently its combination with lapatinib target mainly MCL-1. However, in SKBR3 cells overexpression of BCL-xL partially rescues sensitivity to dinaciclib and its combination with lapatinib (Suplementary Fig. 3B), albeit to a smaller extent than overexpression of MCL-1 does (Fig. 2D). This could be explained by the subsequent binding of the freed BAK to BCL-xL that is supplied exogenously, for which BAK has also affinity^23^. While small molecule BCL-xL inhibitors have so far proven too toxic^49,50^, other strategies to target BCL-xL, for instance, PROTACS, are being developed^51^. Indeed, Brugge and colleagues demonstrated potent preclinical in vivo activity of the dual BCL-xL/BCL-2 inhibitor navitoclax with the HER2-targeting antibody–drug conjugate trastuzumab emtansine^52^.

In contrast to the fairly unknown toxicity of MCL-1 inhibitors, dinaciclib is a CDK1, 2, 5, and 9 inhibitor that has demonstrated limited toxicities as a monotherapy, many of which were transient^6,53^. CDK9 is part of the CAK complex, which is responsible for phosphorylating the C-terminus of RNA polymerase II, regulating elongation during transcription^33^. Although there are other cyclin-dependent kinases that are capable of phosphorylating the CTD of the RNA Polymerase II, like CDK7 and CDK8, the only one that activates gene expression in a catalytic manner is CDK9^54^. CDK9 inhibitors regulate the expression of proteins with a short half-life. In this context dinaciclib has been reported to suppress the expression levels of the homologous recombination (HR) repair factors Rad51 and BRCA1 as well as c-Myc^55,56^. Notwithstanding the fact that MCL-1 is not the only protein that is downregulated after treatment with dinaciclib, the lack of its pro-apoptotic partner, NOXA, in *HER2*-amplified breast cancers^4^ makes it likely the most important dinaciclib target in *HER2*-amplified breast cancers. Of note, there are other CDK inhibitors that have been explored for the treatment of *HER2*-amplified breast cancers, but no correlation with the expression of MCL-1 has been established^57^.

Combining HER2 inhibitors with a targeted therapy that can sensitize to apoptosis is an important therapeutic strategy since a robust apoptosis response is essential for mono-therapeutic targeted therapy in other RTK-driven cancers^58–60^. In fact, in paradigms such as *EGFR*-mutant NSCLC, EGFR inhibition has limited success in patients whose cancers cannot undergo robust apoptosis^58,61–65^. We believe the ability of dinaciclib to rationally combine with HER2 inhibitors to induce apoptosis could therefore overcome the lack of efficacy HER2 inhibitors in *HER2*-amplified breast cancers display, providing a targeted therapy combination strategy that could potentially eliminate the need for chemotherapy.

Since in addition to forming complexes with pro-apoptotic BCL-2 family members, MCL-1 also exerts oncogenic activity through other means^66,67^, pharmaceutical reduction of MCL-1 expression may be more broadly effective than exposure to MCL-1 BH3 mimetics. Indeed, we noted increased sensitivity of dinaciclib and lapatinib compared to A-1210477 and lapatinib (Fig. 1). In addition, it should be noted that both lapatinib and neratinib are considered dual inhibitors of HER2 and EGFR^68,69^, which contributes to dermatologic and gastrointestinal adverse events^70,71^. We also investigated the efficacy of the highly selective HER2 inhibitor tucatinib combined with CDK9 inhibition. Consistently, our data support the notion that combination treatment of dinaciclib with selective HER2 inhibition can be an effective therapy against *HER2*-amplified breast cancer.

In all, we propose that treating *HER2*-positive breast cancers by co-targeting HER2 and MCL-1 can be achieved with the CDK inhibitor dinaciclib, which is clinically advanced. This combination may have advantages over MCL-1 BH3 mimetics, therefore maximizing the potential of HER2 inhibitors to treat *HER2*-amplified breast cancers. Importantly, this offers a strategy that is independent of chemotherapy, with the aim of improving responses and decreasing toxicity.

## Materials and methods

### Cell lines

The *HER2*-positive breast cancer cell lines used in this study were kindly provided by the Massachusetts General Hospital. SKBR3 cells were grown in DMEM/F12 medium with 10% fetal bovine serum (FBS) in the presence of 1 μg/mL penicillin and streptomycin. BT-474 cells were cultured in DMEM medium containing 10% FBS, 1 μg/ml penicillin, streptomycin, and 5 μg/ml of insulin. MDA-MB-453, HCC-1419 were cultured in RPMI with 10% FBS in the presence of 1 μg/mL penicillin and streptomycin. Cells were regularly screened for mycoplasma using a MycoAlert Mycoplasma Detection Kit (Lonza).

### Reagents

The following drugs were purchased: Dinaciclib (SCH727965) for in vitro and in vivo studies (S2768; Selleckchem), lapatinib ditosylate (Tykerb) for in vitro and in vivo *studies* (M1802; Abmole), neratinib for in vivo studies (M1913; Abmole), A-1210477 (CT-A121; Chemietek), A-1331852 (22963; Cayman Chemicals), tucatinib (HY-16069; Medchem), and ABT-199 (venetoclax) (CT-A199; Chemietek). The antibodies used in this study were as follows: Anti-Bak (AB-1 clone for IP) (AM03; EMD Millipore), anti-Bak (3814S; Cell Signaling), anti-Bim (C34C5) (2933S; Cell Signaling), anti–BCL-xL (54H6) (2764S; Cell Signaling), anti–Bcl-2 (D55G8) (Human Specific) (4223S; Cell Signaling), anti-cleaved PARP (Asp214) (D64E10) (5625S; Cell Signaling), anti-GAPDH (6C5) (sc-32233; Santa Cruz), anti–MCL-1 (S-19) (sc-819; Santa Cruz), anti-phospho-p44/42 MAPK (Erk1/2) (Thr202/Tyr204) (D13.14.4E) (4370S; Cell Signaling), anti-phospho-S6 Ribosomal Protein (Ser240/244) (D68F8) (5364S; Cell Signaling), anti-phospho-Akt (Thr308) (244F9) (4056S; Cell Signaling), anti-phospho-Akt (Ser473) (D9E) (4060S; Cell Signaling), anti-HER2/ErbB2 (29D8) (2165S; Cell Signaling), anti-phospho-HER2/ErbB2 (Tyr1248) (2247S; Cell Signaling), anti-phospho-Rpb1 CTD (Ser 2/5) (4375S; Cell Signaling), Normal Rabbit IgG for IP (sc-2027; Santa Cruz), and Normal Mouse IgG for IP (sc-2025; Santa Cruz).

### Vector construction and establishing stable cell lines

For the short-hairpin RNA (shRNA) experiments, the lentiviral shRNA (shBAK) was purchased from Open Biosystems. shRNA designed against a scramble sequence (MISSION pLKO.1-shRNA control plasmid DNA) served as the control. The pLKO.1 puromycin-resistant vector backbone served as the basis for cell selection in puromycin following infection. Cells were transduced with plasmid containing viral particles that were generated in 293T cells and collected over 48 h. The human MCL1 expression vector was generated as previously described (2). The construct was transfected into 293T packaging cells along with the packaging plasmids and the lentivirus-containing supernatants were collected to transduce the cells.

### Western blotting

Cell lines and tumors from BT-474 xenografts as well as PDXs were prepared and lysed in lysis buffer (20 mM Tris, 150 mM NaCl, 1% Nonidet P-40, 1 mM EDTA, 1 mM EGTA, 10% glycerol, and protease, and phosphatase inhibitors), incubated on ice for 15 min, and centrifuged at max speed for 10 min at 4 °C. Tumor lysates were homogenized with Tissuemiser (Fisher Scientific) in the lysis buffer described previously, incubated for 20 min on ice, and centrifuged at max speed for 10 min at 4 °C. Equal amounts of the detergent-soluble lysates were resolved using the NuPAGE Novex Midi Gel system on 4–12% Bis–Tris gels (Invitrogen), transferred to polyvinylidene fluoride membranes (PerkinElmer) in between six pieces of Whatman paper (Fisher Scientific) set in transfer buffer from Biorad with 20% methanol, and following transfer and blocking in 5% nonfat milk in PBS, probed overnight with the antibodies listed above. Representative blots from at least three independent experiments are shown in the figures. Chemiluminescence was detected with the Syngene G: Box camera (Synoptics).

### Cell viability assay

For the Cell Titer-Glo experiments, 1000–3000 seeded cells per well in 96-well flat-bottom black plates were treated with 25 μL of CellTiter-Glo (Promega), following continuous drug treatment (each time with the indicated drugs at the indicated concentrations), at 37° and 5% atmospheric CO_2_ and immediately read on a Centro LB 960 microplate luminometer (Berthold Technologies) according to the Promega protocol. Quantification of no-treatment seeded cells was used to determine the total cell growth number over the experiment. All data are means ± SD of three independent experiments (*n* = 3).

### FACS apoptosis assay

Totally, 3 × 10^5^ cells were seeded per well in six-well plates and drugged with 100 nM dinaciclib combined with 1 μM lapatinib for 24 (BT-474) and 72 h (MDA-MB-453), or left untreated. Cells were incubated with propidium iodide and annexin V-Cy5 (BD Biosciences) together for 15 min and assayed on a Guava easyCyte 5 flow cytometer (Millipore Sigma). Analysis was performed using guavaSoft 3.1.1 software. Cells stained positive for annexin V and annexin V + propidium iodide were counted as apoptotic. All data are means ± SD of three independent experiments (*n* = 3).

### RNA extraction and qRT-PCR

RNA was isolated from cultured cells grown at sub-confluency using the Zymo Quick-RNA MiniPrep kit (Zymo Research), and RNA was reverse-transcribed to form cDNA molecules using cDNA synthesis kit superscript III (Invitrogen) on a 7500 Fast Real-Time PCR System (Life Technologies). The expression of *MCL-1*, and *β-ACTIN* (*ACTB*) was measured using a GENEAMP PCR System 9700 (Life Technologies) by measuring the fluorescence increases of SYBR Green (Roche). The primers for *MCL-1* forward 5′-GGGCAGGATTGTGACTCTCATT-3′ and *MCL-1* reverse 5′-GATGCAGCTTTCTTGGTTTATGG-3′ and for *ACTB* forward 5′-GGCATGGGTCAGAAGGATT-3′, and *ACTB* reverse 5′-AGGATGCCTCTCTTGCTCTG-3′. To determine relative abundance of *MCL-1* in relation to *ACTB*, the Delta-Delta CT (cycle threshold) method was utilized. All data are means  + SEM of three independent experiments (*n* = 3).

### Immunoprecipitation

Cells were lysed in the same buffer above; 500 μg of lysates were incubated each time with MCL-1 antibody (2000 ng), or rabbit IgG (2000 ng). Following the addition of 25 μL of 1:1 PBS: prewashed Protein A Sepharose CL-4B beads (cat. no. 17–096303; GE Healthcare Life Sciences) to the antibody/lysate mix, samples were incubated with rotating motion overnight. Equal amounts of extracts (5% of immunoprecipitated protein) were also prepared. Representative blots from at least three independent experiments are shown in the figures. Chemiluminescence was detected with the Syngene G: Box camera (Synoptics).

### BAK activation assay

Cells were treated as indicated and lysed in AB-1 amino terminal capture buffer (10 mM Hepes,135 mM NaCl, 5 mM MgCl_2_, 0.2 mM EDTA,1% glycerol + 1% CHAPS, added fresh; pH 7.4); 1500 μg of lysates for the assay were incubated each time with AB-1/BAK antibody (1000 ng). Following the addition of 25 μL of 1:1 PBS: prewashed Protein A Sepharose CL-4B beads (cat. no. 17-0963-03; GE Healthcare Life Sciences) to the antibody/lysate mix, samples were incubated with rotating motion overnight. Equal amounts of extracts (2.5% of immunoprecipitated protein) were also prepared. Representative blots from at least three independent experiments are shown in the figures. Chemiluminescence was detected with the Syngene G: Box camera (Synoptics).

### Xenograft studies

NSG female mice were injected with ∼15 × 10^6^ BT-474 cells per 200 μL of 1:1 (cells: Matrigel). Mice were injected intraductally both sides and monitored for tumor growth. When tumors reached ∼200 mm^3^, the tumor-bearing mice were randomized to a no-treatment control group, a lapatinib group (100 mg/kg), a dinaciclib group (40 mg/kg), or a combination group (same doses). Mice in the cohorts (control = 4 tumors, lapatinib = 5 tumors, dinaciclib = 4 tumors, combination = 4 tumors) were treated with dinaciclib via IP injection and 2 h later with lapatinib by oral gavage. The solvent for lapatinib was 1% Tween 80. Dinaciclib was formulated in 20% 2-hydroxy propyl-β-cyclo dextrin (Sigma-Aldrich). The tumors were measured daily by electronic caliper, in two dimensions (length and width), and with the formula *v* = l × (*w*)2(*π*/6), where *v* is the tumor volume, *l* is the length, and *w* is the width (the smaller of the two measurements). The drug schedule was 5 days a week (Monday–Friday) for lapatinib and twice a week for dinaciclib for 30 days. For pharmacodynamic studies, tumors were harvested 2 h following the last lapatinib treatment, and tumors were snap frozen in liquid nitrogen. All mouse experiments were approved and performed in accordance with the Institutional Animal Care and Use Committee at VCU.

### Patient-derived xenografts

Female NSG mice were inoculated with tumor pieces derived from two HER2 + breast cancer PDX models called WHIM 8 and WHIM 22 (Horizon Discovery Group^35^, expanded as single cell suspensions and injected into experimental mice orthotopically at the amount indicated in the legend of Fig. 4. Tumor growth was monitored until tumors grew to treatable levels (∼150 mm^3^). These mice were then randomized into four groups: control, neratinib (40 mg/kg), dinaciclib (40 mg/kg), and dinaciclib/neratinib combination treatment. The number of tumors per cohort was: control = 4 tumors, neratinib = 4 tumors, dinaciclib = 4 tumors, combination = 4 tumors for the WHIM 22 model and control = 5 tumors, neratinib = 5 tumors, dinaciclib = 5 tumors, combination = 5 tumors for the WHIM 8 model. Dinaciclib was formulated in 20% 2-hydroxy propyl-β-cyclo dextrin (Sigma-Aldrich), while the solvent for neratinib was 0.5% methocellulose—0.4% Tween 80. Mice in the cohorts were treated with dinaciclib via IP injection and 2 h later with neratinib by oral gavage. The drug schedule was 5 days a week (Monday–Friday) for neratinib and twice a week for dinaciclib for 16 days (WHIM 22) or 18 days (WHIM 8). For pharmacodynamic studies, tumors were harvested 2 h following the last neratinib treatment, and tumors were snap frozen in liquid nitrogen. Tumors were measured as per the BT-474 xenograft.

### Statistical considerations

Two-tailed Student’s *t* test was performed for Figs. 1B–D, 2D, F, 3B, D, Suplementary Fig. 1, Suplementary Fig. 3B, D, Suplementary Fig. 4 and Suplementary Fig. 5A–C using GraphPad Prism. *p* values were corrected for multiple testing using Bonferroni method. For Figs. 4A, 5A and 5C one-way Anova test was performed for comparisons between lapatinib/neratinib, dinaciclib and combination cohorts. Dunnett’s test was used as post hoc. Differences were considered statistically different if *p* < 0.05. A *p* value < 0.05 is indicated by *, *p* < 0.01 by **, *p* < 0.001 by ***, and *p* < 0.0001 by ****.

## Supplementary information

Supplemental Figure Legends

Figure S1

Figure S2

Figure S3

Figure S4

Figure S5
