# Supplementary material for: Targeting transcription of MCL-1 sensitizes HER2-amplified breast cancers to HER2 inhibitors
Source: Cell Death Dis. 2021 Feb 15;12(2):179. doi: 10.1038/s41419-021-03457-6 (PMC7884408; doi:10.1038/s41419-021-03457-6)
Supplement: Supplementary file 1 — Supplemental Figure Legends [file 41419_2021_3457_MOESM1_ESM.docx]

**Supplemental Figure Legends**

**Figure S1.** Dinaciclib sensitizes *HER2*-amplified breast cancer cells to lapatinib and is superior to the MCL-1 BH3 mimetic A-1210477. **(A)** BT-474 cells were treated with increasing concentrations of lapatinib in combination with 10 μM A-1210477 (left) and 100 nM dinaciclib (right) for 24h and cell viability was determined by Celltiter-Glo. n=3; error bars indicate ±SD. **(B)** BT-474 cells were treated with increasing concentrations of A-1210477 (left) or dinaciclib (right) in combination with 1 μM lapatinib for 24h and cell viability was detected. n=3; error bars indicate ±SD. **(C)** and **(D)** same as **(A)** and **(B)** treating MDA-MB-453 cells for 72h. n=3; error bars indicate ±SD. For Figs. S1A, S1B, S1C and S1D two-tailed Student’s *t* test was performed; p-values were corrected for multiple testing using Bonferroni method. Differences were considered statistically different if p < 0.05. A p-value < 0.05 is indicated by *, p< 0.01 by **, p < 0.001 by ***, and p < 0.0001 by ****.

**Figure S2.** Dinaciclib combined with lapatinib induces apoptosis in *HER2*-amplified breast cancer cells. FACS analysis showing annexin-V-Cy5 and propidium iodide staining in BT-474 **(A)** and MDA-MB-453 **(B)** cells following treatment with 1 μm lapatinib in combination with 100 nM dinaciclib for 24 and 72h respectively. ‘’No Rx’’: No drug.

**Figure S3.** Dinaciclib functions mainly by inhibiting MCL-1. **(A)** SKBR3 control and BCL-2 or BCL-xL-expressing cells were treated with 1 μM lapatinib, 100 nM dinaciclib and their combination for 12h. Whole cell lysates were prepared, subjected to western blotting and probed for the indicated proteins. **(B)** SKBR3 control and BCL-2 or BCL-xL-expressing cells were treated with 1 μM lapatinib, 100 nM dinaciclib and their combination for 12h and subjected to CellTiter-Glo. n=3; error bars indicate ±SD. **(C)** MDA-MB-453 control and BCL-2 or BCL-xL-expressing cells were treated with 1 μM lapatinib, 100 nM dinaciclib and their combination for 12h. Whole cell lysates were prepared, subjected to western blotting, and probed for the indicated proteins. **(D)** MDA-MB-453 control and BCL-2 or BCL-xL-expressing cells were treated with 1 μM lapatinib, 100 nM dinaciclib and their combination for 72h and subjected to CellTiter-Glo. n=3; error bars indicate ±SD. For figures S3B, S3D two-tailed Student’s *t* test was performed. p-values were corrected for multiple testing using the Bonferroni method. A p-value < 0.05 is indicated by *, p< 0.01 by **, p < 0.001 by ***, and p < 0.0001 by ****. EV: Empty Vector, ‘’No Rx’’: No drug.

**Figure S4.** BCL-2 pro-survival proteins other than MCL-1 are not significantly suppressed in dinaciclib-induced apoptosis in *HER2*-amplified breast cancer cells. **(A)** BT-474 cells were treated with increasing concentrations of lapatinib in combination with 1 μM ABT-199 (venetoclax), 1μM A-1331852 and 10 μM A-1210477 for 24h and cell viability was determined by Celltiter-Glo. **(B)** BT-474 cells were treated with increasing concentrations of dinaciclib in combination with 1 μM ABT-199 (venetoclax), 1μM A-1331852 and 10 μM A-1210477 for 24h and cell viability was evaluated. **(C)** and **(D)** same as **(A)** and **(B)** treating MDA-MB-453 cells for 72h. n=3; error bars indicate ±SD for Sup. Fig. 4A – Sup Fig. 4D. Two-tailed Student’s *t* test was performed for figures S4A-S4D; p-values were corrected for multiple testing using Bonferroni method. Differences were considered statistically different if p < 0.05. A p-value < 0.05 is indicated by *, p< 0.01 by **, p < 0.001 by ***, and p < 0.0001 by ****.

**Figure S5.** Combination treatment of dinaciclib with lapatinib and neratinib leads to anti-tumor activity *in vivo*. **(A)** BT-474 breast cancer cells were grown as xenograft tumors in female NSG mice, and when tumors were ∼150 mm^3^, mice were randomized into treatment cohorts (control, lapatinib, dinaciclib and combination) as described in material and methods and treated for 30 days before harvesting the tumors. Tumor measurements were performed every other day, and the average tumor volume + SEM for each cohort is displayed. Asterisks indicate a significant separation between the combination (lapatinib/dinaciclib) and dinaciclib (orange) or the combination and lapatinib (green) treatment cohorts using the Student’s *t* test. * p < 0.05, ** p < 0.01, *** p < 0.001, **** p < 0.0001. **(B)** WHIM 22 PDX breast cancer cells were injected orthotopically into each NSG mouse, and when tumors reached the size of 150–200 mm^3^, mice were randomized into treatment cohorts (control, neratinib, dinaciclib, combination) as described in material and methods and treated for 16 days before harvesting the tumors. Tumor measurements were performed every other day, and the average tumor volume + SEM for each cohort is displayed. Asterisks indicate a significant separation between the combination (neratinib/dinaciclib) and dinaciclib (orange), or the combination and neratinib (green) treatment cohorts using the Student’s *t* test. * p < 0.05, ** p < 0.01, *** p < 0.001, **** p < 0.0001. **(C)** same as **(B)** using WHIM 8 PDX breast cancer cells. The WHIM 8 PDX injected mice were treated for 18 days before harvesting the tumors.
