# Supplementary figures and images for: Targeting transcription of MCL-1 sensitizes HER2-amplified breast cancers to HER2 inhibitors
Source: Cell Death Dis. 2021 Feb 15;12(2):179. doi: 10.1038/s41419-021-03457-6 (PMC7884408; doi:10.1038/s41419-021-03457-6)

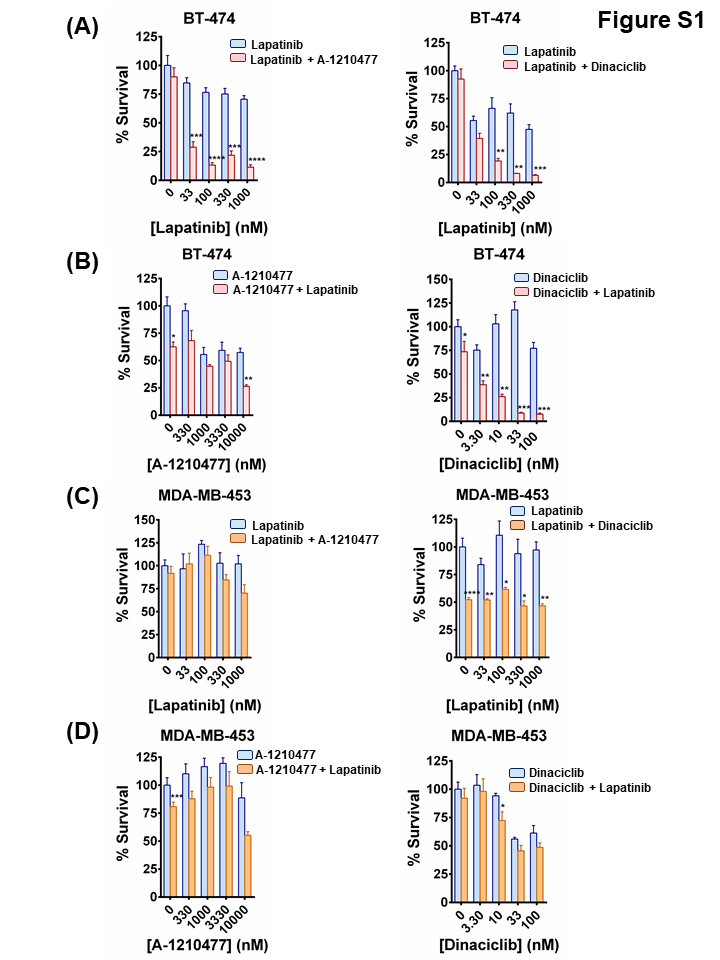

Supplement: Supplementary file 2 — Figure S1 [file 41419_2021_3457_MOESM2_ESM.tif]

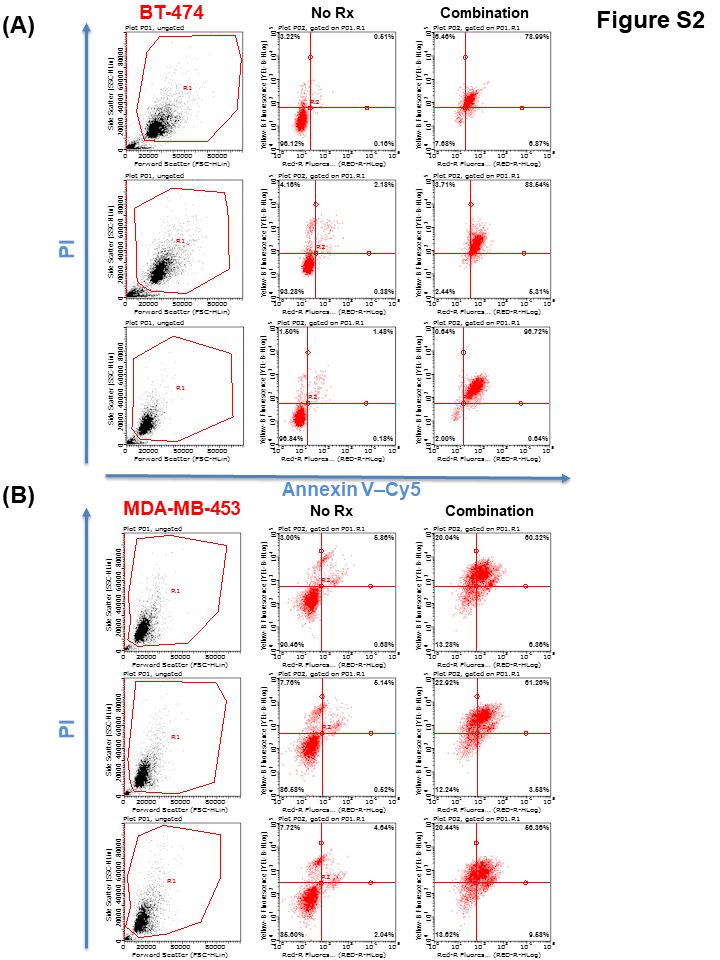

Supplement: Supplementary file 3 — Figure S2 [file 41419_2021_3457_MOESM3_ESM.tif]

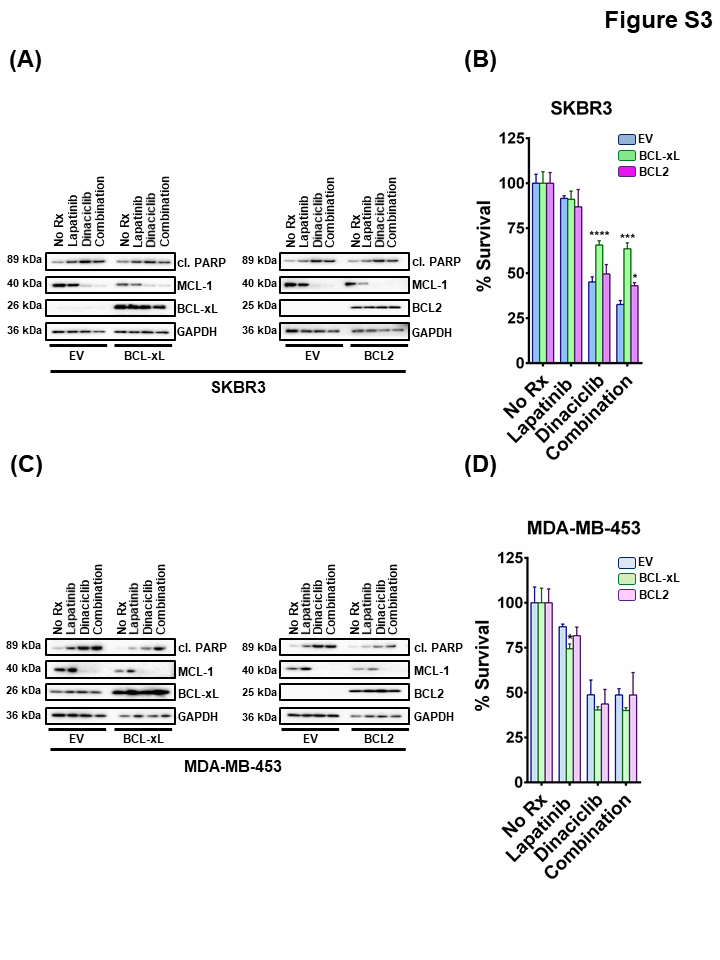

Supplement: Supplementary file 4 — Figure S3 [file 41419_2021_3457_MOESM4_ESM.tif]

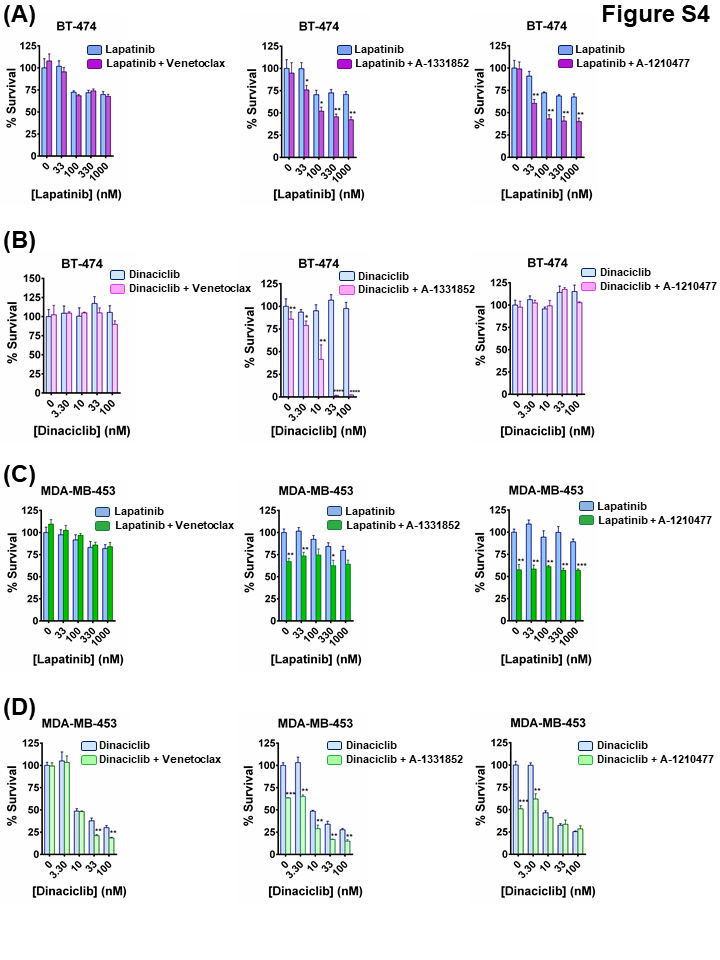

Supplement: Supplementary file 5 — Figure S4 [file 41419_2021_3457_MOESM5_ESM.tif]

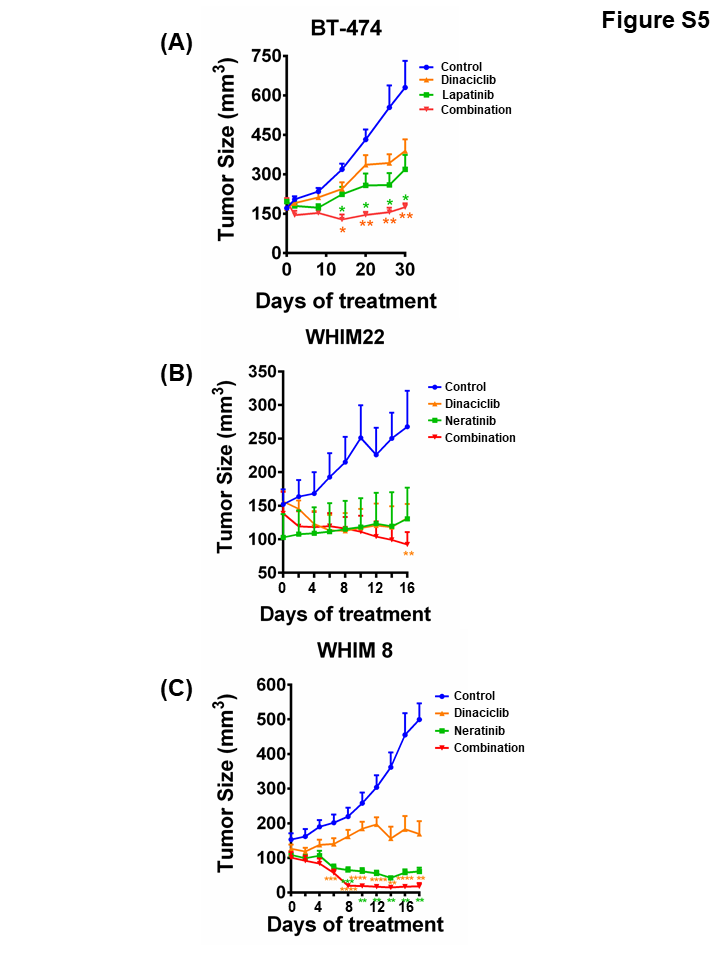

Supplement: Supplementary file 6 — Figure S5 [file 41419_2021_3457_MOESM6_ESM.tif]
